# Neonatal 6-OHDA lesion model in mouse induces Attention-Deficit/ Hyperactivity Disorder (ADHD)-like behaviour

**DOI:** 10.1038/s41598-018-33778-0

**Published:** 2018-10-18

**Authors:** Otmane Bouchatta, Houria Manouze, Rabia Bouali-benazzouz, Nóra Kerekes, Saadia Ba-M’hamed, Pascal Fossat, Marc Landry, Mohamed Bennis

**Affiliations:** 1 Laboratory of Pharmacology, Neurobiology and Behavior (URAC-37), Faculty of Sciences, Cadi Ayyad University, Marrakesh, Morocco; 2 Bordeaux University, Bordeaux, France; 3 Interdisciplinary Institute of Neuroscience, CNRS UMR 5297 Centre Paul Broca-Nouvelle Aquitaine, Bordeaux, France; 4 Department of Health Sciences, University West, Trollhättan, Sweden

## Abstract

Attention-deficit/hyperactivity disorder (ADHD) is a common neurodevelopmental disorder characterized by impaired attention, impulsivity and hyperactivity. The “neonatal 6-hydroxydopamine” (6-OHDA) lesion is a commonly used model of ADHD in rat. However, a comprehensive assessment of ADHD‐like symptoms is still missing, and data in mouse remain largely unavailable. Our aim was to analyse symptoms of ADHD in the mouse neonatal 6‐OHDA model. 6‐OHDA mice exhibited the major ADHD‐like symptoms, i.e. hyperactivity (open field), attention deficit and impulsivity (five‐choice serial reaction time task). Further, the model revealed discrete co‐existing symptoms, i.e. anxiety‐like (elevated plus maze test) and antisocial (social interaction) behaviours and decreased cognitive functioning (novel object recognition). The efficacy of methylphenidate, a classical psychostimulant used in the treatment of ADHD, was also evaluated. A histological analysis further supports the model validity by indicating dopamine depletion, changes in cortical thickness and abnormalities in anterior cingulate cortex neurons. A principal component analysis of the behaviour profile confirms that the 6‐OHDA mouse model displayed good face and predictive validity. We conclude that neonatal dopamine depletion results in behavioural and morphological changes similar to those seen in patients and therefore could be used as a model for studying ADHD pathophysiological mechanisms and identifying therapeutic targets.

## Introduction

Attention-deficit/hyperactivity disorder (ADHD) is a heterogeneous neurodevelopmental psychiatric disorder that affects 5–8% of children and can persist at adulthood in 50% of cases^1,2^. Three core clinical symptoms define ADHD: hyperactivity, inattention and impulsivity^3^. ADHD patients also often coexisting complains such as poor academic performance, social disabilities and emotional deficits^4,5^.

In line with possible dysfunctions in the networks involved in controlling and regulating attention and/or impulsivity^6^, neuropsychological studies consistently demonstrated impaired prefrontal cortex (PFC) functions in ADHD patients correlated with reduced PFC size, blood flow and glucose metabolism^7^. Moreover, ADHD patients have impaired dopamine (DA) and norepinephrine (NE) transmission that affects PFC function^8^. Consequently, the primary drugs used for treating ADHD enhance DA and NE transmission. Psychostimulants (e.g. methylphenidate, d-amphetamine, and pemoline) are the most commonly used pharmacological treatments and target dopaminergic system. ADHD treatments remain however poorly efficient and are accompanied with side effects^9^. Thus, a better understanding of ADHD neurochemical mechanisms is key to improve treatments and valid animal models are required for this purpose.

Ideally a reliable animal model should display all the symptoms present in ADHD patients, and respond similarly to the same pharmacological interventions^10^. Several animal models of ADHD currently exist but none were developed specifically for modeling neurodevelopmental alterations that occur during the disease onset and progression, neither to model several aspects of the behavioral and executive functional symptoms. The most widely used model of ADHD is the inbred spontaneously hypertensive rat (SHR). Besides, the most classical neurodevelopmental model of ADHD created by lesioning brain systems is obtained by neonatal 6-hydroxydopamine (6-OHDA) injection. In this model, studies mainly focused on hyperactivity symptoms^10–12^. However, data regarding impulsive behavior or attention deficits remain unclear^13^.

The aim of the present study is to evaluate the repertoire of major and comorbid symptoms of ADHD in a 6-OHDA lesioned mouse model. We evaluated face validity through assessing spontaneous activity during juvenile period, coexisting complains in adolescence and the persistence of ADHD-like symptoms at the adulthood. The efficacy of methylphenidate, a classical psychostimulant acting as a catecholamine transporter inhibitor and used in the treatment of ADHD, was also used to test predictive validity of the model. We further analyzed specific morphological characteristics of ADHD in our model, focusing on PFC, particularly anterior cingulate cortex (ACC) neurons. Finally, a principal component analysis of the behavior profile demonstrates the importance of individual symptoms in the pathology.

## Results

### 6-OHDA injection at P5 depletes dopamine immunoreactivity in adult mice

**Figure 1:**
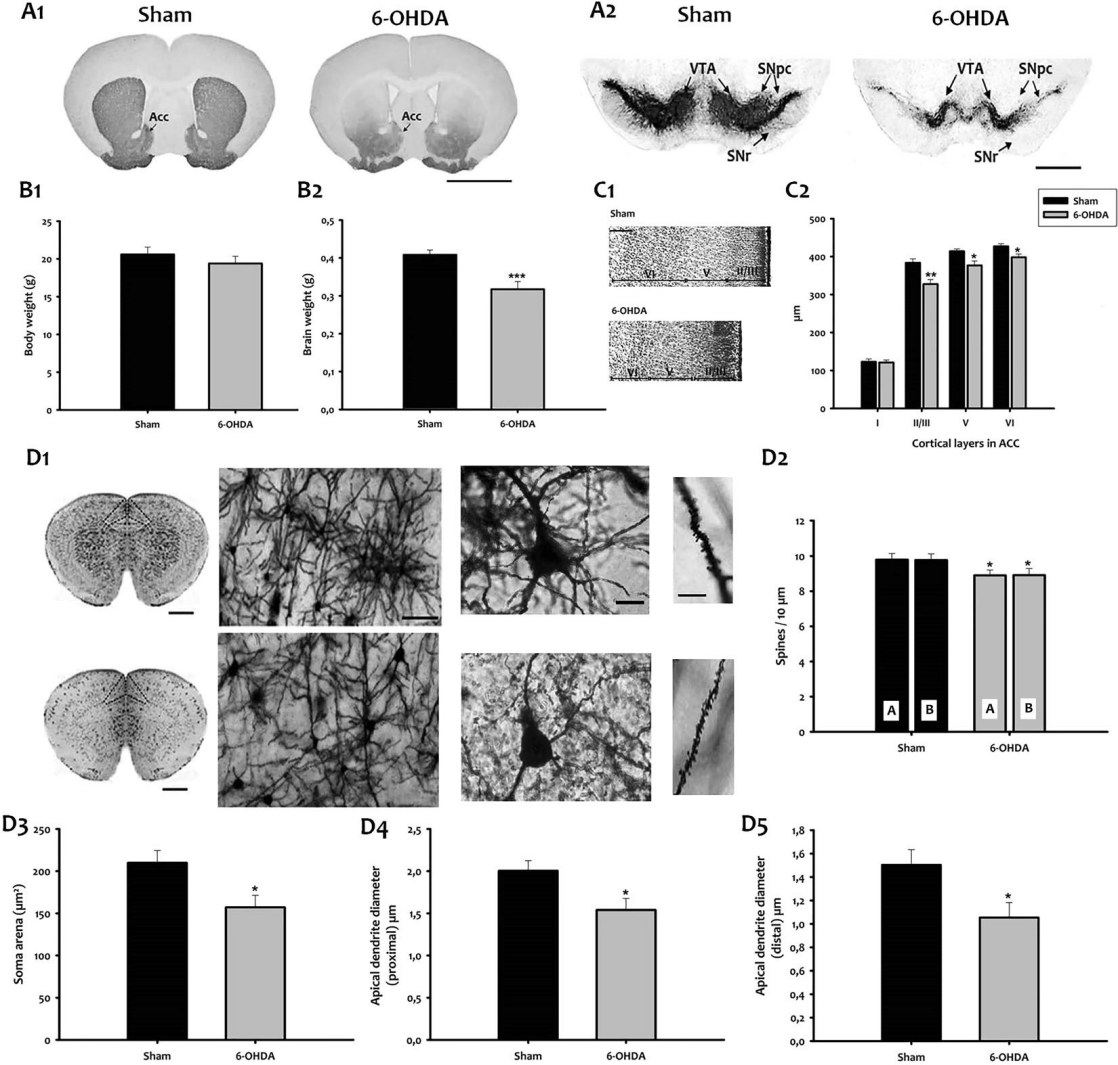
Morphological characteristics of 6-OHDA mice. TH immunohistochemistry in the striatum (**A1**), and ventral midbrain (**A2**) of adult mice. Bar = 1000 μm. (**B1**,**B2)** Body and brain weight in grams. (**C1)**. Nissl staining of the ACC. Bar = 400 μm. (**C2**). Thickness of cortical layers from I to VI (μm). Values are represented as mean ± SEM (*p < *0*.*05*; **p < *0*.*01*; ***p < *0*.*001*; t-test; n = 7). (**D1)** (Left) Representative micrographs of mouse brains stained with Golgi Cox. Bar = 1000 μm. (Center left) Higher magnification images of Golgi-Cox stained brain sections from sham (top) and 6-OHDA (down) mice are shown. Bar = 100 μm. (Center right) Golgi staining of layer II-III pyramidal neurons (ACd) in sham (top) and 6-OHDA mice (down). Bar = 15 μm. (Right) Micrographs of Golgi-stained neurons showing dendrites of sham (top) and 6-OHDA (down). Bar = 25 μm. (**D2)** Dendritic spine density of apical and basal dendrites in the ACd neurons of the investigated experimental groups. A/B, apical/basal dendrites. (**D3**) Area of neuronal cell bodies. **D4** Apical dendrite diameter (proximal) at 10 μm from the soma. (**D5**) Apical dendrite diameter (distal) at 100 μm from the soma. Bars represent mean ± SEM (*p < *0*.*05*). Acc, accumbens nucleus; ACd, dorsal anterior cingulate cortex; SN, substantia nigra; SNpc, SN pars compacta; SNr, SN pars  reticulata; VTA, ventral tegmental area.

To examine whether 6-OHDA mice display dopamine depletion, we examined TH-immunoreactivity (IR) in the striatum and midbrain of sham and 6-OHDA adult mice. We found a high density of TH-IR fibers in the striatum of sham mice (Fig. 1A1). In contrast, the TH content was significantly reduced in the striatum of 6-OHDA mice (Fig. 1A1). Similarly, a dense TH-IR was observed in substantia nigra (SN) and ventral tegmental area (VTA) of sham mice (Fig. 1A2), while 6-OHDA resulted in severe, bilateral loss of TH-IR in SN and VTA (Fig. 1A2).

### 6-OHDA injection induces cortical alteration

We then evaluated body and brain weight in 6-OHDA mice and sham (Fig. 1B1–2**)**. No significant difference was seen in body weight between the two groups (Fig. 1B1). However, compared to sham mice (0.48 g ± 0.01, n = 10), 6-OHDA mice (0.32 g ± 0.02, n = 9) showed 25% reduction in brain weight (*p* < *0*.*001*; Fig. 1B2). Next, we assessed cortical thickness with Nissl staining that revealed a significant reduction in cortical layers II-III, V and VI in 6-OHDA mice compared to sham (Fig. 1C1–2; layer II-III, *p* < *0*.*01*; layer V, *p* < *0*.*05*; and layer VI, *p* < *0*.*05*).

Since the dorsal anterior cingulate cortex (ACd) is one of the main targets involved in the modulation of attention and executive functions, we investigated the morphology of layer II-III pyramidal neurons of the ACd using Golgi staining (Fig. 1D1). Qualitative abnormalities of dendritic branching were noted in various regions of Golgi-Cox stained cortical tissue from 6-OHDA mice. Although not quantified, observations at high magnification indicated a lower complexity in branching of medium-sized ACd neurons (Fig. 1D1), and an apparent decrease in the size of dendritic spines (Fig. 1D1). The mean spine density on the apical (A) and basal (B) dendrites of layer III ACd pyramidal neurons is significantly reduced in 6-OHDA animals as compared to shams (Fig. 1D2; n = 10 neurons/animal, n = 3 mice/group; in total n = 30 neurons/group; *p* < *0*.*05*). In 6-OHDA mice, the area of pyramidal neurons is smaller than in sham (Fig. 1D3; *p* < *0*.*05*). Moreover, the diameter of apical dendrites of the layer II-III pyramidal neurons at 10 μm (proximal) and 100 μm (distal) from the soma showed a significant reduction in 6-OHDA mice at both distance (Fig. 1D4–5; *p* < *0*.*05*).

Collectively, these data indicate that 6-OHDA mice exhibited the morphological characteristics of ADHD, including dopamine depletion, changes in cortical thickness and abnormalities in ACd neurons.

### 6-OHDA juvenile mice exhibit hyperactivity

In order to characterize the ADHD symptoms, we first investigated the spontaneous locomotor activity of 6-OHDA and sham mice in an open field at p24.

**Figure 2:**
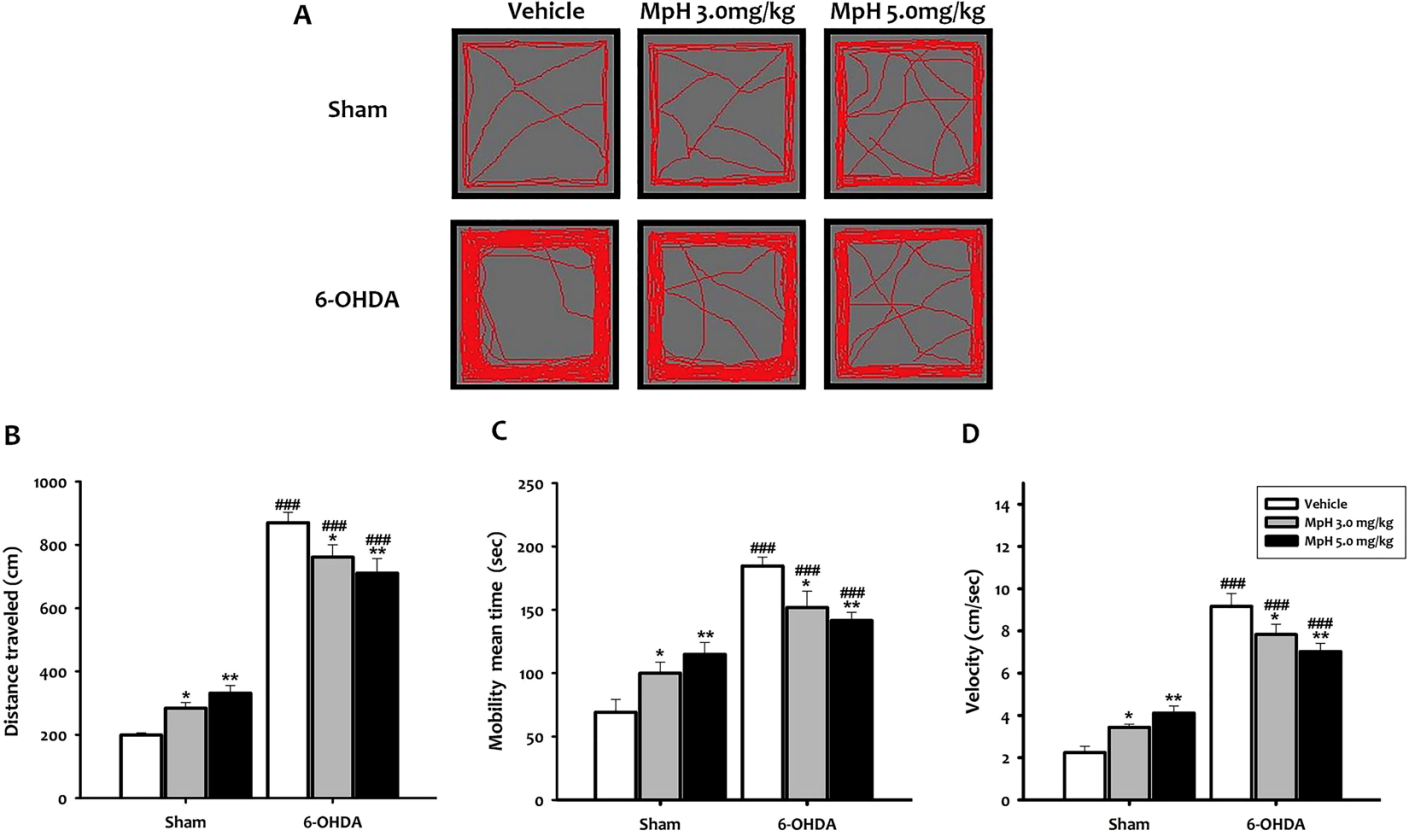
Spontaneous locomotor activity at P24. (**A)**. Representative activity traces in the open field. 6-OHDA mice presented higher locomotor activity than sham animals. (**B**) Distance traveled during 10 min. (**C)** Mobility mean time. (**D**) Animal velocity. All data shown are means ± SEM, n = 10 mice per group, *p < *0*.*05* and **p < *0*.*01 vs* vehicle; ^###^*p* < *0*.*001 vs* sham.

Two-way ANOVA analysis was performed with lesion and treatment as main factors. It showed that the distance traveled, mobility mean time and speed of animal movement are significantly affected by the lesion (F_(1,54)_ = 411.40; F_(1,54)_ = 71.95 and F_(1,54)_ = 205.57, *p* < *0*.*001;* respectively), the treatment (F_(2,54)_ = 40.11; F_(2,54)_ = 32.66 and F_(2,54)_ = 57.59, *p* < *0*.*05;* respectively) and the interaction between lesion and treatment (F_(2,54)_ = 11.67; F_(2,54)_ = 11.67 and F_(2,54)_ = 12.57, *p* < *0*.*001;* respectively). At P24, Student-Newman-Keuls *post-hoc* analysis showed that 6-OHDA mice presented higher locomotor activity than shams (Fig. 2A) with a significantly longer distance traveled (q = 21.83, *p* < *0*.*001*; Fig. 2B), as well as a higher mobility mean time (q = 12.37, *p* < *0*.*001*; Fig. 2C) and speed of animal movement (q = 17.10, *p* < *0*.*001*; Fig. 2D). These observations supported hyperactivity in 6-OHDA mice at P24.

Moreover, we evaluated the capacity of Mph to alleviate hyperactivity. The injection of 3.0 or 5.0 mg/kg Mph dose-dependently decreased the mean distance travelled (q = 3.53, *p* < *0*.*05*; q = 5.18, *p* < *0*.*01*; respectively; Fig. 2A,B), mobility mean time (q = 3.51, *p* < *0*.*05*; q = 4.61, *p* < *0*.*01*; respectively; Fig. 2C) and velocity (q = 3.29, *p* < *0*.*05*; *p* < *0*.*01*; respectively; Fig. 2D) as compared to vehicle-injected 6-OHDA mice. The opposite effect of Mph was observed in sham mice. Importantly, we found that spontaneous locomotor activity remained increased in P40 adolescent mice (Fig. S1).

After demonstrating that hyperactivity, one of the major symptoms, is phenocopied in the 6-OHDA mouse model, we focused on coexisting complains of the syndrome, namely anxiety, antisocial behavior and cognitive impairments in adolescent mice (see Material and Methods).

### 6-OHDA adolescent mice show anxiety-like behavior

**Figure 3:**
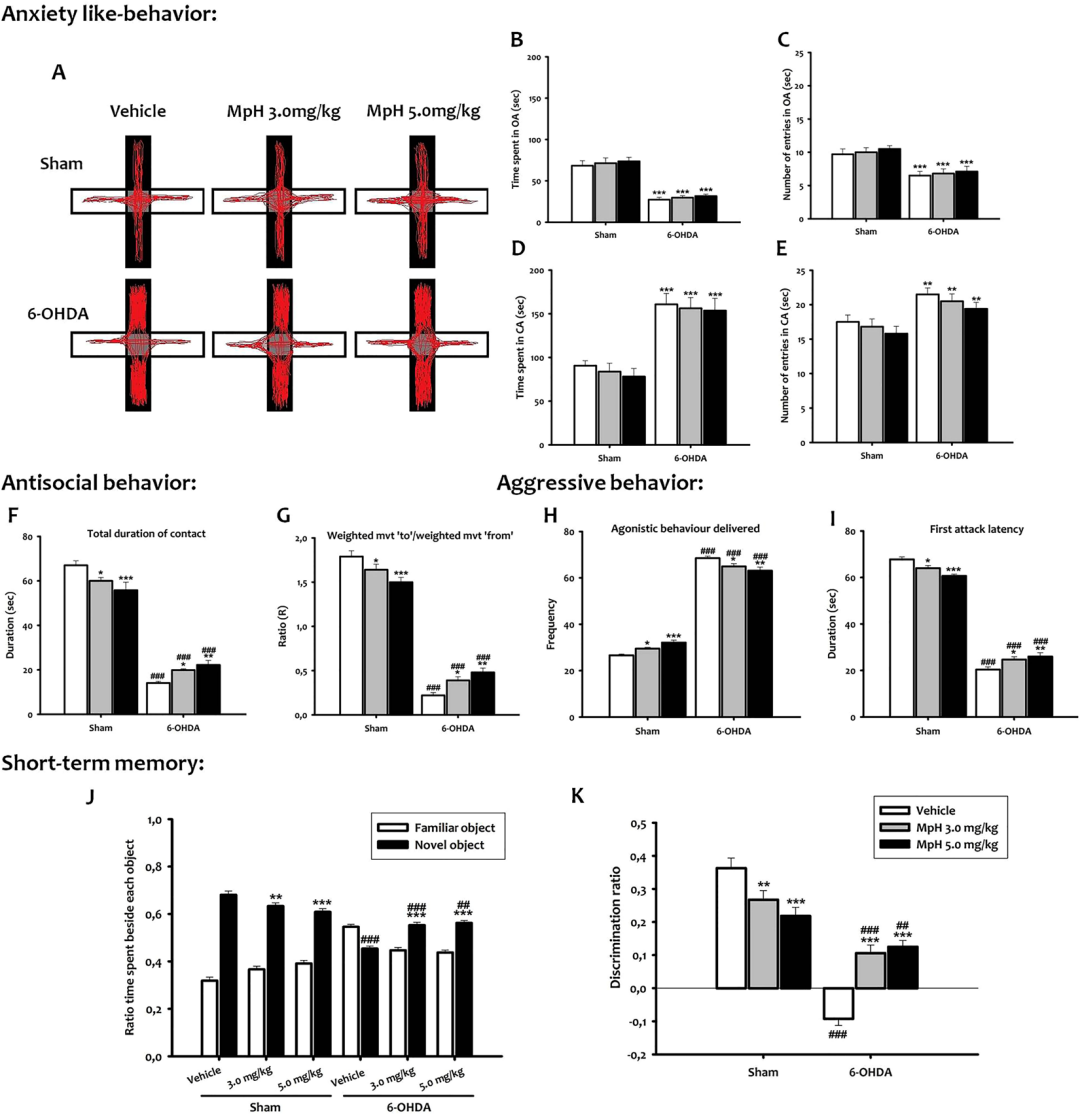
Anxiety like-behavior (**A**–**E**), antisocial interaction (**F**,**G**), aggression (**H**,**I**) and memory impairment (**J**,**K**) of 6-OHDA mice. (**A**) Video-tracking data illustrating reluctance of a representative 6-OHDA mouse to explore the arms of the EPM relative to sham. (**B**) Time spent in EPM open arm. (**C**) Number of entries in EPM open arm. (**D**) Time spent in EPM closed arm. (**E**) Number of entries in EPM closed arm expressed by the two groups respectively. (**F**) Total duration of contacts. (**G**) Ratio between the weighted movement to and the weighted movement from the paired mouse. (**H**) Agonistic behavior delivered. I. First attack latency. (**J**) Time ratio (Tr) spent on each object. (**K**) Discrimination ratio (Dr). All data shown are means ± SEM, n = 10 mice per group, **p < *0*.*01* and ***p < *0*.*001 vs* vehicle; ^##^*p* < *0*.*01* and ^###^*p* < *0*.*001 vs* sham.

A two way ANOVA confirmed a main effect of lesion in all anxiety parameters (time spent and number of entries in the open and closed arms) (F_(1,54)_ = 20.40; F_(1,54)_ = 33.55; F_(1,54)_ = 67.24; F_(1,54)_ = 132.23, *p* < *0*.*001*; respectively), whereas the treatment (F_(2,54)_ = 1.73; F_(2,54)_ = 0.52; F_(2,54)_ = 0.40; F_(2,54)_ = 0.61, *p* > *0*.*05*; respectively) and the interaction treatment/lesion (F_(2,54)_ = 0.021; F_(2,54)_ = 0.014; F_(2,54)_ = 0.03; F_(2,54)_ = 0.007, *p* > *0*.*05*; respectively) did not affect the parameters. In the elevated plus-maze (EPM) test, the time spent in the open arms (q = 9.26, *p* < *0*.*001*) and the number of entries (q = 4.63, *p* < *0*.*001*) were reduced in the 6-OHDA group as compared to the sham group (Fig. 3A–C). However, 6-OHDA mice entered more frequently in the closed arms of the maze (q = 3.91, *p* < *0*.*01*; Fig. 3A,E), where they spent significantly longer times than sham mice (q = 6.46, *p* < *0*.*001*; Fig. 3D). Neither 3.0 mg/kg nor 5.0 mg/kg Mph influenced the time spent by 6-OHDA mice in open (q = 0.54 and q = 1.00, *p* > *0*.*05;* respectively; Fig. 3A,B) or closed arms (q = 0.42 and q = 0.66, *p* > *0*.*05*; respectively; Fig. 3A,D). No Mph effect was seen in sham mice as compared to vehicle-treated animals either in open (q = 0.70 and q = 1.22, *p* > *0*.*05*; respectively; Fig. 3B) or in closed arms (q = 0.62 and q = 1.14, *p* > *0*.*05*; respectively; Fig. 3D).

### 6-OHDA adolescent mice exhibit antisocial behavior

The social interaction test measured the levels of social anxiety based on animal’s interactions. Two-way ANOVA, with lesion and treatment as main factors, revealed that the antisocial behavior (avoidance intensity and total duration of contact) was significantly affected by the lesion (F_(1,54)_ = 915.87; F_(1,54)_ = 644.06, *p* < *0*.*001*; respectively), treatment (F_(2,54)_ = 12.71; F_(2,54)_ = 30.58, *p* < *0*.*05*; respectively) and the interaction treatment/lesion (F_(2,54)_ = 14.22; F_(2,54)_ = 11.67, *p* < *0*.*001*; respectively). The *post hoc* analysis showed that the sham animals approached the control animals, whereas the 6-OHDA animals avoided them (q = 30.31, *p* < *0*.*001*; Fig. 3G). Both 3.0 mg/kg and 5.0 mg/kg Mph increased the approach behavior in 6-OHDA mice (q = 3.28, *p* < *0*.*05*; q = 5.01, *p* < *0*.*01*; respectively), whereas the same doses increased the avoidance behavior in sham mice (q = 2.89, *p* < *0*.*05;* q = 5.59, *p* < *0*.*001*; respectively; Fig. 3G). Also, during a social interaction test in a novel environment, the total duration of contacts of 6-OHDA mice was significantly lower than sham mice (q = 26.00, *p* < *0*.*001;* Fig. 3F). By contrast, Mph treatment increased these parameters in 6-OHDA mice (q = 2.89, *p* < *0*.*05*; q = 3.99, *p* < *0*.*01*; respectively) as compared to vehicle-injected 6-OHDA mice, while the opposite was seen in sham mice (q = 3.44, *p* < *0*.*05*; q = 5.50, *p* < *0*.*001*; respectively; Fig. 3F).

Interestingly, we also found that 6-OHDA mice exhibited not only antisocial, but even aggressive behavior. Indeed, two-way ANOVA analysis with lesion and treatment as main factors, revealed that both agonistic behavior and first attack latency were significantly affected by lesion (F_(1,54)_ = 2209.82; F_(1,54)_ = 1726.65, *p* < *0*.*001*; respectively), treatment (F_(2,54)_ = 91.50; F_(2,54)_ = 88.51; *p* < *0*.*05*; respectively) and the interaction treatment/lesion (F_(2,54)_ = 12.25; F_(2,54_ = 14.53, *p* < *0*.*001*; respectively). 6-OHDA mice displayed a significant increase of the agonistic behavior (number of biting attacks) against a stimulus mouse as compared to sham mice (q = 43.86, *p* < *0*.*001*; Fig. 3H). In addition, the first attack latency was significantly lower in 6-OHDA mice than in sham mice (q = 39.74, *p* < *0*.*001*; Fig. 3I). Both Mph doses decreased significantly those parameters in the 6-OHDA mice (Mph 3.0 mg/kg: q = 3.87, *p* < *0*.*05;* q = 3.60; *p* < *0*.*05* and Mph 5.0 mg/kg: q = 3.76, *p* < *0*.*01;* q = 4.69, *p* < *0*.*01*, respectively). The same treatment has an opposite effect in sham mice (Mph 3.0 mg/kg: q = 3.03, *p* < *0*.*05;* q = 3.18, *p* < *0*.*05* and Mph 5.0 mg/kg: q = 5.75, *p* < *0*.*001;* q = 5.95; *p* < *0*.*001*; respectively; Fig. 3H–I).

### Impairment of short-term memory in 6-OHDA adolescent mice

In the present study, we assessed also cognitive functions through memory and learning. Object recognition was ascertained by greater time interacting with the novel than the familiar object (assessed by the ratio of time spent, Tr), and a discrimination ratio (Dr) above 0.5 (see suppl. methods) (Fig. 3J,K).

Two way ANOVA analysis with lesion and treatment as factors indicated that the Tr and Dr were affected significantly by the lesion (F_(1,54)_ = 200.74 and F_(1,54)_ = 131.70, *p* < *0*.*001*), the treatment (F_(2,54)_ = 25.21 and F_(2,54)_ = 19.20, *p* < *0*.*05*) and the interaction treatment/lesion (F_(2,54)_ = 14.53 and F_(2,54)_ = 28.11; *p* < *0*.*001*). In the novel object recognition test, the exploration time of a new object was significantly reduced in 6-OHDA mice (q = 18.03, *p* < *0*.*001*; Fig. 3J) compared to sham mice. Moreover, the Dr was significantly lower in the 6-OHDA group as compared to sham group (q = 18.02, *p* < *0*.*001*; Fig. 3K), suggesting that the cognitive abilities and recognition memory may also be impaired in 6-OHDA mice. Mph at 3.0 mg/kg or 5.0 mg/kg increased the exploration time of the new object in 6-OHDA mice (q = 7.85, *p* < *0*.*001* and q = 8.61, *p* < *0*.*001*; respectively), whereas reduced in sham mice (q = 3.78, *p* < *0*.*01* and q = 5.72, *p* < *0*.*001*; respectively; Fig. 3J). Additionally, Mph improved the Dr in 6-OHDA mice (q = 7.85, *p* < *0*.*001* and q = 8.61, *p* < *0*.*001*; respectively), while the opposite was seen in sham mice (q = 3.78, *p* < *0*.*001* and q = 5.72, *p* < *0*.*001*; respectively; Fig. 3K).

Interestingly, we found that 6-OHDA adolescent mice showed an increase in anxiety, antisocial and aggressive behaviors, and deficits in learning and memory system, which are the discrete symptoms of ADHD. In addition, Mph improved cognitive malfunctions, enhances social interactions, but has no effect in anxiety-like behavior.

### 6-OHDA adult mice show attention deficit and impulsivity

Next, we analyzed the two other major symptoms of ADHD, inattention and impulsivity, with the 5-CSRTT test.

Two-way ANOVA repeated measures with lesion and session as main factors, indicated that the accuracy, omission, premature response and perseverative responding in the baseline 5-CSRTT performance were significantly affected by lesion (F_(1, 27)_ = 156.62; F_(1, 27)_ = 360.00; F_(1, 27)_ = 228.60; F_(1, 27)_ = 305.01, *p* < *0*.*001;* respectively), while the session (F_(3,27)_ = 0.034; F_(3,27)_ = 0.11; F_(3,27)_ = 1.21; F_(3,27)_ = 4.42, *p* > *0*.*05;* respectively) and the interaction lesion/session (F_(3,27)_ = 0.017; F_(3,27)_ = 0.017; F_(3,27)_ = 2.39; F_(3,27)_ = 6.94, *p* > *0*.*05;* respectively) had no effect. During the acquisition phases of the last 4 sessions of training at 1 s of SD, 6-OHDA mice were less accurate than sham mice (t = 6.22, *p* < *0*.*001*; Fig. S2A), made more omission (t = 8.00, *p* < *0*.*001*; Fig. S2B), premature (t = 5.10, *p* < *0*.*001*; Fig. S2C) and perseverative responses (t = 6.19, *p* < *0*.*001*; Fig. S2D). Thus, our data suggest that attentiveness is impaired and impulsivity increased in 6-OHDA mice.

To test that possibility, we manipulated the inter-trial interval (ITI) and the stimulus duration (SD) to evaluate impulsivity and attention, respectively, when mouse performances remained stable.

#### Impulsivity test

**Figure 4:**
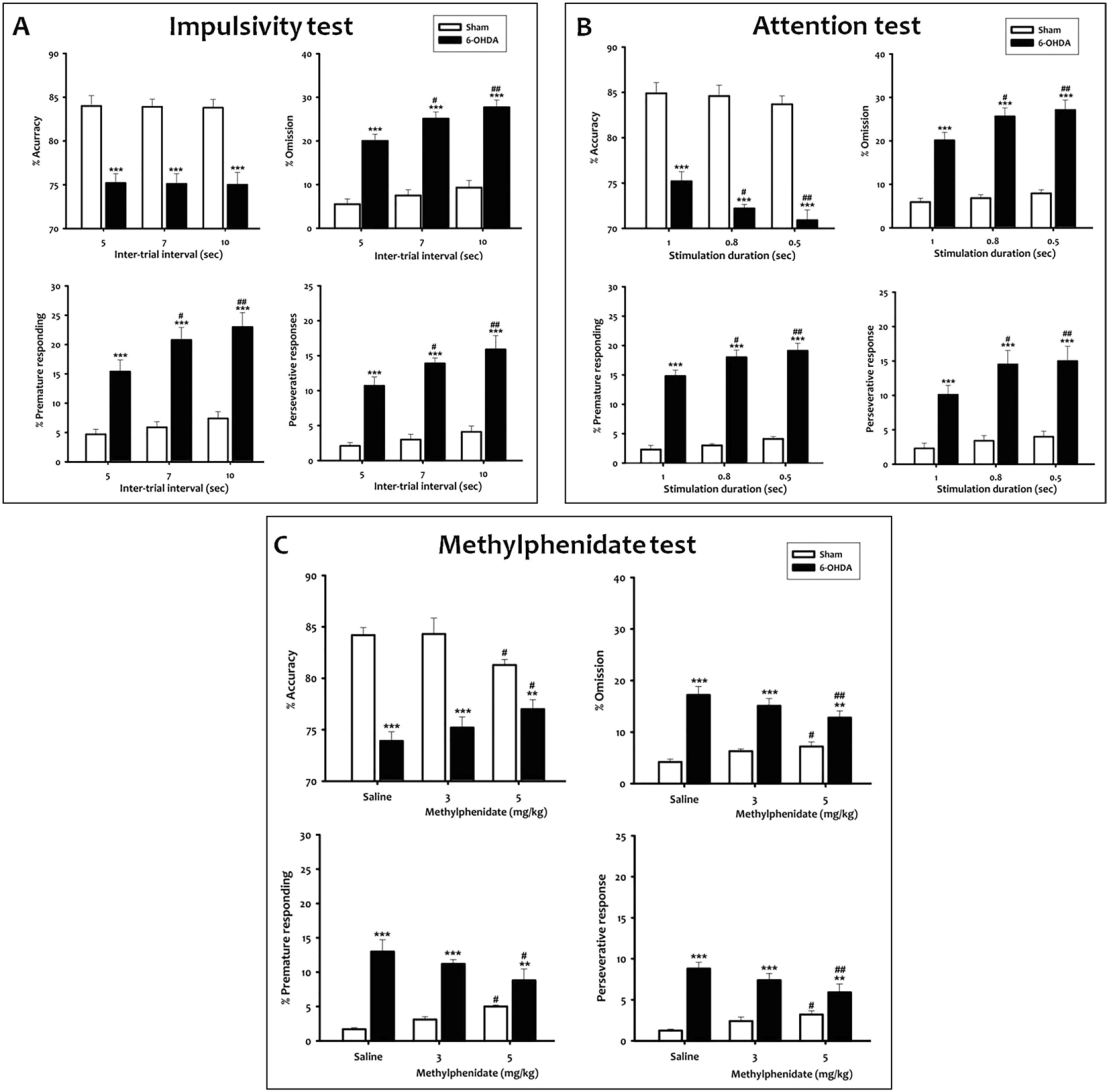
Impulsivity and inattention of 6-OHDA mice in the 5-CSRTT. (**A**) Inter-trial interval (ITI) test in the 5-CSRTT. (**A**) significant increase in premature responding, perseverative responding and omissions was observed when the ITI was lengthened from 5 to 7 or 10 sec in 6-OHDA mice. (**B**) Stimulus duration (SD) test in the 5-CSRTT. (**A**) significant decrease in accuracy and an increase in omissions, premature response and perseverative responses were observed in 6-OHDA mice, when the SD was decreased from 1 to 0.8 or 0.5 s. (**C**) Effect of the drug treatment in the 5-CSRTT. Mph at 5.0 mg/kg produced a significant increase in accuracy, and a decrease in omissions, premature responding, and perseverative responses in 6-OHDA mice. Data represent percentage of accuracy, omissions, premature responding and perseverative responses. Data is expressed as mean ± SEM, n = 10 mice per group, **p < *0*.*01* and ***p < *0*.*001 vs* sham; ^#^*p* < *0*.*05* and ^##^*p* < *0*.*01 vs* vehicle.

Two-way ANOVA repeated measures with lesion and (ITI) as main factors, showed a significant effect of lesion (F_(1, 18)_ = 45.64, *p* < *0*.*001*), while the ITI and the interaction lesion/ITI had no effect on accuracy parameter (F_(2,18)_ = 0.015, *p* > *0*.*05;* F_(2,18)_ = 3.51, *p* > *0*.*05;* respectively). Furthermore, the statistical analysis indicated a significant effect of lesion (F_(1, 18)_ = 183.84; F_(1, 18)_ = 85.65 and F_(1, 18)_ = 205.96, *p* < *0*.*001;* respectively) and ITI (F_(2,18)_ = 5.13, *p* < *0*.*01*; F_(2,18)_ = 5.28, *p* < *0*.*01*; F_(2,18)_ = 4.34, *p* < *0*.*05;* respectively) on the omission, premature and preservative responses. In contrast, the interaction lesion/ITI had no effect on those parameters (F_(2,18)_ = 1.27; F_(2,18)_ = 1.42; F_(2,18)_ = 1.39, *p* > *0*.*05;* respectively). When the ITI was lengthened from 5 to 7 or 10 sec, a significant increase in premature (t = 2.40, *p* < 0.05; t = 3.38, *p* < *0*.*01;* respectively), perseverative (t = 2.03, *p* < *0*.*05*; t = 3.30, *p* < 0.01*;* respectively), and omissions (t = 2.30, *p* < 0.05; t = 3.46, *p* < *0*.*01;* respectively) responses was observed in 6-OHDA mice **(**Fig. 4A**)**. This suggests that 6-OHDA mice impulsivity was disclosed by ITI increase. Meanwhile, we did not observe difference on accuracy in 6-OHDA mice when the ITI increased (t = 0.07 or t = 0.14, *p* > *0*.*05*; Fig. 4A). No change in these parameters was observed in the sham group.

#### Attention test

Two way ANOVA repeated measures indicated significant effects of lesion on all parameters (accuracy: F_(1,18)_ = 185.52; omission: F_(1,18)_ = 172.02; premature responding:F_(1,18)_ = 743.99 and perseverative response: F_(1,18)_ = 37.62, *p* < 0.001) and the SD (F_(2,18)_ = 3.11; F_(2,18)_ = 3.46; F_(2,18)_ = 6.82 and F_(2,18)_ = 4.74, *p* < 0.05; respectively), whereas, the interaction lesion/SD had no effect (F_(2,18)_ = 1.51; F_(2,18)_ = 2.00; F_(2,18)_ = 1.03 and F_(2,18)_ = 1.23, *p* > *0*.*05*; respectively). In fact, when SD decreased from 1 to 0.8 or 0.5 s, the *post-hoc* analysis indicated a significant decrease in accuracy (t = 2.04, *p* < *0*.*05* and *t* = 2.92, *p* < *0*.*01*) and an increase in omissions (t = 2.46, *p* < *0*.*05* and *t* = 3.12, *p* < *0*.*01*) in 6-OHDA mice (Fig. 4B). We also observed a significant effect of the 6-OHDA lesion on premature (t = 2.45, *p* < *0*.*05* and t = 3.29, *p* < *0*.*01*) and perseverative responses (t = 2.65, *p* < *0*.*05* and *t* = 2.95, *p* < *0*.*01*) (Fig. 4B). No effect was observed in the sham group.

#### Methylphenidate test

Mice were subjected to standard sessions of the 5-CSRTT with the same parameters as for the assessment of baseline responding. For all parameters (accuracy, omission, premature responding and perseverative response), there is a significant effect of lesion (F_(1,18)_ = 116.99; F_(1,18)_ = 50.04; F_(1,18)_ = 126.28; F_(1,18)_ = 58.36, *p* < *0*.*001;* respectively), treatment (F_(2,18)_ = 23.15; F_(2,18)_ = 35.40; F_(2,18)_ = 50.25; F_(2,18)_ = 74.25, *p* < *0*.*01*; respectively) and the interaction treatment/lesion (F_(2,18)_ = 6.95, *p* < *0*.*01*; F_(2,18)_ = 6.97, *p* < *0*.*01*; F_(2,18)_ = 5.46, *p* < *0*.*05*; F_(2,18)_ = 6.76, *p* < *0*.*01*; respectively). In 6-OHDA mice, Mph at 5.0 mg/kg produced a significant increase in accuracy (t = 2.21, *p* < *0*.*05*), a decrease in omissions (t = 3.16, *p* < *0*.*01*), premature (t = 2.85, *p* < *0*.*05*), and perseverative responses (t = 3.02, *p* < *0*.*01*; Fig. 4C) as compared to vehicle-injected 6-OHDA mice. By contrast, in sham mice, Mph at the same dose induced a significant decrease in accuracy (t = 2.14, *p* < *0*.*05*), an increase in omissions (t = 2.15, *p* < *0*.*05*), premature (t = 2.03, *p* < *0*.*05*), and perseverative responses (t = 2.04, *p* < *0*.*05*; Fig. 4C) as compared to vehicle-injected 6-OHDA mice. Meanwhile, we did not observe a significant effect of Mph at 3.0 mg/kg in 6-OHDA or sham groups.

#### PCA analysis of 6-OHDA induced ADHD model in mice

**Figure 5:**
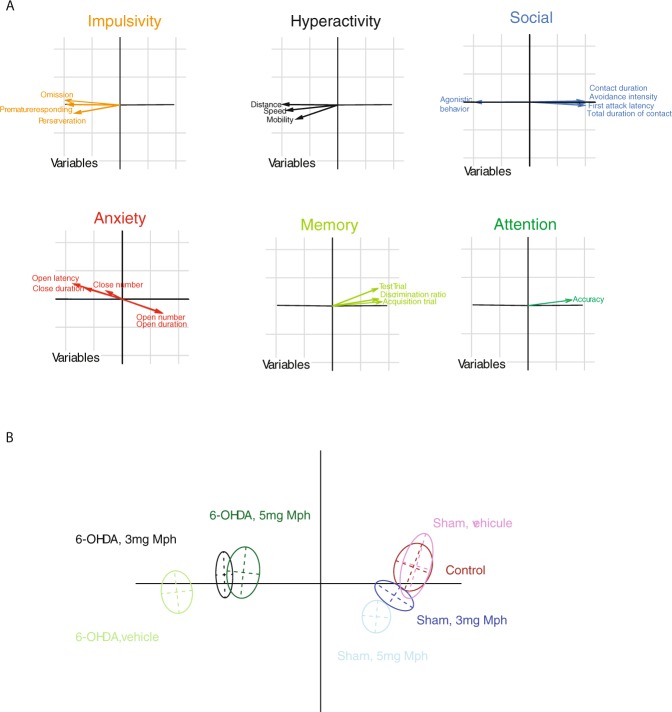
Principal component analysis (PCA) of the 20 behavioral variables measured in experimental groups.

To compare all experimental groups, we performed principal component analysis (PCA) based on the 20 variables measured in the different behavioral tests (see methods). The PCA enabled the representation of the variables (Fig. 5A) and experimental groups (Fig. 5B) on two orthogonal axis. The first component (x-axis) is composed of almost all behavioral variables with a major action of variables associated with social aggression, impulsivity and hyperactivity (see table S1 and Fig. 5A, Comp 1 in bold). Control and sham groups are not different (Figs 5B and S3A1 and A2, p = 1, Monte-Carlo) but significantly separated from the 6-OHDA group along the x-axis (Figs 5B and S3A1 and A2, p = 0.001, Monte-Carlo). The second component (y-axis) is composed of variables linked to anxiety and memory impairments (see table S1 and Fig. 5A, Comp 2 in bold). Control, sham and 6-OHDA are not clearly separated along this axis (Fig. 5B). This indicated that the 6-OHDA mouse model is a good model to analyze ADHD symptoms related to the variables that composed the x-axis i.e aggression, hyperactivity and impulsivity (Fig. 5B). Mph significantly reduced the symptoms along the x-axis, but not the y-axis (Figs 5B, S3B1 and B2). Finally, in control groups, Mph has opposite effects, significantly modifying group values along the y-axis (Fig. S3C1 and C2, p < 0.001, Monte-Carlo). Therefore, Mph may favour anxiety and memory impairments in control.

## Discussion

The diagnostic of ADHD, like other psychiatric disorders, relies on behavioural assessment. Animal models of ADHD must mimic clinical symptomatology and in particular the three core symptoms of hyperactivity, impulsivity, and impaired attention^8,14^, but also other comorbid affections.

Here we provide evidence that the 6-OHDA mouse model exhibits known major symptoms of the human pathology, namely hyperactivity in a novel environment at a juvenile stage, inattention and impulsive-like behavior at adulthood. Moreover, the 6-OHDA adolescent mouse exhibits co-morbid symptoms including increased anxiety, antisocial and aggressive behaviors, and deficits in learning and memory. We also discuss below the mechanisms underlying dopamine depletion-induced pathophysiology and we point to the interest of the 6-OHDA model in mimicking the effects of known treatments.

### The 6-OHDA mouse model exhibits known symptoms of the human pathology

Disrupting brain systems through neonatal 6-OHDA lesion is a classical neurodevelopmental model of ADHD in rat^11,15^. Selective removal of DA projections to forebrain in neonatal rats leads to age-limited spontaneous motor hyperactivity^16–18^ at an age corresponding to human periadolescence^11,19^. Only one study used such a model in mouse and reproduced similar locomotor impairment^12^. However, data regarding impulsive behavior or attention deficits remain unavailable in mouse^13^, or contradictory in rat^10,15,20^. Moreover, existing studies do not describe discrete co-morbid symptoms.

ADHD adult patients show inattention and elevated impulsivity^21^ that can be illustrated by ADHD patient performance of the continuous performance task^22,23^. ADHD subjects have slower and more variable reaction times, and make more errors of omission indicative of poor attentional ability^21^. In addition, they make more errors of commission, demonstrating reduced behavioral inhibition and impulsivity. In our study, hyperactivity is determined in 6-OHDA mice with the open field test. One of our main findings was that 6-OHDA adult mice displayed deficits in inhibitory control in the 5-CSRTT, a task used in rodents that requires behavioral inhibition^24^. 6-OHDA mice exhibit an increased number of perseverative responding under baseline conditions and increased premature responding during the inter-trial interval challenge. Impulsive choice reflects, to a greater degree, decision-making processes rather than motoric inhibition^25^. This is generally considered to reflect a failure of the «executive system» represented by frontal cortical areas exerting a top-down control to limbic and paralimbic areas^26^.

Likewise, 6-OHDA mice displayed a greater loss of accuracy when attention was challenged. Interestingly, this effect was present all along the session, indicative of a deficit in selective attention and difficulty to maintain sustained attention. Taken together, these data demonstrate that 6-OHDA adult mice exhibit attention deficit and impulsivity.

Beside the major symptoms, ADHD children exhibit cognitive impairments and short-term memory deficits^5,27,28^. Access to novelty (e.g. object or environment) can elicit approach behaviors in rodents. Starting from this observation^29^, a new behavioral test was developed in the late 1980s: the so-called object recognition test^30^. The test is based on the rodent tendency to interact more with a novel than a familiar object. The exploration time of a new object during the test trial was not significantly increased in 6-OHDA mice, suggesting that cognitive abilities (e.g. learning) and/or recognition memory were impaired in 6-OHDA adolescent mice. These findings are in agreement with previous studies showing cognitive impairments in spatial discrimination task in rats^10^ and in mice^12^. Anxiety disorder is also a common comorbidity of ADHD^27^ that we explored with the EPM test. 6-OHDA adolescent mice exhibited anxiety-like behavior in agreement with previous results^12^. Another set of discrete symptoms of ADHD is characterized by aggression and disruptive behavior^31–34^. We found that 6-OHDA adolescent mouse showed an antisocial behavior, including reduced social interaction and aggressive attitude.

### Differences with other existing animal models of ADHD

Very few animal models of ADHD have been able to mimic multiple deficits at the same time. In the early 1960s, the spontaneously hypertensive rat (SHR) was developed by inbreeding Wistar-Kyoto (WKY) rats^35^. SHR shows several major ADHD-like symptoms such as hyperactivity^36^, impulsivity and poor attention^37^. However, SHR rats also show hypertension that is not reported in patients with ADHD^14^, thus making difficult to disassociate the effects of the two disorders. Indeed, hypertension is a potential confounding factor for SHR as a model for ADHD, suggesting that altered norepinephrine (NE) transmission^38,39^ may contribute to hypertension rather than hyperactivity^10^. Moreover, the WKY control often shows low activity levels, and has even been suggested as a model of depression^40–42^.

Another genetic model is the mouse strain lacking the dopamine transporter (DAT KO)^43^. DAT KO mice show hyperlocomotion in novel environment^43^ and impaired learning and memory^44^. However, DAT KO mice display extremely elevated dopamine levels in the striatum and nucleus accumbens^45^ unlike ADHD patients^46,47^. Moreover, testing this model predictive validity with psychostimulants is impossible because of the absence of the DAT protein, the primary target of these drugs^48^. Other mouse genetic models have been proposed but lack face and/or predictive validity, e.g. the Coloboma Mutant Mouse^49^.

### Mechanisms of dopamine depletion-induced pathology

Hyperactivity in human subjects with ADHD is accompanied by decreased dopamine in striatum, prefrontal cortex, septum, midbrain and amygdala^46,50,51^. Furthermore, multiple lines of evidence recently supported the view that neuroanatomical alterations exist in ADHD patients. Studies have reported decreased brain volume in patients with ADHD, slowed maturation and reduced connectivity in the prefrontal cortex, anterior cingulate cortex, basal ganglia, and cerebellum^13^. In particular, the dorsal cingulate cortex, which plays a key role in the modulation of attention and executive functions^52^, appears to be dysfunctional in patients with ADHD^4^. Moreover, lateral prefrontal development in children with ADHD is delayed by several years^53^, and the anterior cingulate cortex is thinner in adults with ADHD^54^. Studies using functional magnetic resonance imaging in ADHD children sitting still or performing a continuous task also showed smaller sizes of DA target areas, including the prefrontal cortex and striatum and deficits in the basal ganglia^55^. Our data demonstrate that 6-OHDA mouse model of ADHD also exhibits such anatomical characteristics of ADHD, including decreased in brain weight and volume brain, smaller sizes of DA target areas, cortical thickness and abnormalities in dorsal anterior cingulate neurons.

### Predictive validity of the 6-OHDA mouse model

Animal models of ADHD should be capable of predicting therapeutic effects in patients. Human studies have shown that Mph increases impulse control^56^, attention^57^, and working memory^58^ in ADHD patients. Comparable findings have been obtained in rats^59^. We show here that therapeutic-equivalent doses of Mph effectively attenuate the ADHD-like symptoms in 6-OHDA mouse model. Mph reduces hyperactivity of 6-OHDA mice. Moreover, pre-treatment with Mph 30 min before testing 6-OHDA mice in 5-CSRTT resulted in increased attention. The PCA also confirms that Mph is most effective on both the major and discrete symptoms. In contrast, excessive DA stimulation might cause PFC dysfunction leading to impaired inhibition of undesirable behavior, and a deficit in sustaining attention^60^. This can explain the opposite effects of Mph observed in sham group.

### Limits of the model

Mice are nocturnal animals and displayed their main activity peak at the beginning of the dark phase. They have more locomotor activity during night time than they would during the day. However, in the literature, most protocols use a normal and non-inverse light–dark cycle. All behavioral tasks were performed here at the same period (morning or afternoon) for all animals used to avert any circadian related fluctuation in the performance of the animals.

Neurochemical lesion studies have been able to assign specific roles in the regulation of behaviour to discrete brain areas, circuits and neurotransmitter systems, and to mirror the effects of dysfunctional anatomical loci as seen in humans with ADHD^61^. However, ADHD symptoms are the result of several dysfunctional loci interacting within a neural network to produce observable behaviours. Nevertheless, 6-OHDA lesion, although artificial, has comparable behavioural consequences and hence has important implications for research on the specific hypothetical construct^61^. The fact that so many of the ADHD symptoms can be simulated highlights the possibility that the syndrome may have multiple aetiologies, which however may impinge on common neural systems. These systems respond to a common pharmacological treatment, e.g. psychomotor stimulants. Hence, it is possible that the 6-OHDA mouse model reproduces the symptoms of the human disease through the dysfunction of this common target system of the pharmacological treatment.

Because psychostimulants, which increase catecholamine neurotransmission, have been the primary ADHD treatment for decades, clinicians and researchers conjectured that hypodopaminergic function is the neurobiological mechanism underlying ADHD. However, a feature of the 6-OHDA rat model of ADHD is the hyperactivity of the remaining dopaminergic system, and changes in dopamine receptor expression and function, even if the overal effect is a decrease of dopaminergic transmission^62–65^. This reveals a profound compensation and attempts to maintain homeostasis in the residual dopamine terminals in adulthood.

While both dopamine and norepinephrine are known to regulate motor activity, attention, learning, and cognition, dopamine has been the focus of ADHD research. In the neonatal 6-OHDA-lesioned rat model, selective dopamine depletion is achieved by pretreatment with desipramine, which protects noradrenergic nerves. This model illustrates that dopamine depletion alone is sufficient to produce ADHD-like behaviors^66^. Behavioral studies using noradrenergic drugs on animal models indicate that norepinephrine transmission does, indeed, affect ADHD symptoms, but the outcomes are mixed. Enhancing norepinephrine transmission by blocking the NET improves hyperactivity in neonatal 6-OHDA-lesioned rats^15^. From these studies, one cannot infer a causal relationship between increasing/decreasing norepinephrine neurotransmission and severity of ADHD symptoms. Instead, these studies suggest that norepinephrine has dual effects on ADHD-like behaviors^61^.

Currently no serotonergic medications are prescribed in the treatment of ADHD. most studies using animal models of ADHD suggest that serotonin acts to compensate for aberrant dopamine and/or norepinephrine signaling. 6-OHDA-lesioned rats exhibit serotonergic hyperinnervation and the elimination of serotonergic hyperinnervation by administration of the selective serotonergic toxin 5,7-DHT greatly potentiates hyperactivity^63^. Conversely, an increase in serotonergic transmission via serotonin agonist m-CPP or SERT blocker, fluoxetine, greatly reduces hyperactivity^67,68^.

## Conclusion

Although animal models created by the use of neurotoxins do not inform about the causes of ADHD, they are useful tools for studying the contribution of specific brain areas or circuits to cognitive processes that are affected by this pathology^69^. The use of 6-OHDA animal models of ADHD can inform on the cognitive functions sub-served by the lesioned area. It aids to a better understanding of this disorder by parceling the ADHD syndrome into distinct comorbid deficits and provides a valid and suitable animal model for pharmacological tests. We demonstrated in the present study that neonatal 6-OHDA mouse model of ADHD is a valid and reliable model for pre-clinical studies on this neurodevelopmental disorder.

## Material and Methods

### Animals

We used 60 *Swiss* male mice, bred in the central animal facility of Cadi Ayyad University, Marrakech, Morocco. After birth, mice (weighing 4 g) were housed with their mothers in litters and kept under constant temperature conditions (22 °C ± 2), using a 12 h light/12 h dark cycle (lights on at 7 am), with water and food *ad libitum*. The study received approval of the Council Committee of research laboratories of the Faculty of Sciences, Cadi Ayyad University. All procedures were conducted in accordance with the approved institutional protocols and within the provisions for animal care and use prescribed in the scientific procedures on living animals, European Council Directive (EU2010/63). All efforts were made to minimize any animal suffering.

### 6-OHDA neonatal lesion at P5

Intracerebroventricular injection of 6-OHDA was performed at P5 following published protocol^12^. Pups received 6-OHDA hydrobromide (Sigma-Aldrich, France) or vehicle in one of the lateral ventricles after desipramine hydrochloride pretreatment (Sigma-Aldrich, France), under hypothermal anesthesia. The site of injection was set at 0.6 mm lateral to the medial sagittal suture, 2 mm rostral to the lambda and 1.3 mm in depth from the skull surface. 60 mice (P5) were divided on Sham and 6-OHDA groups (n = 30/each group). Each experimental animals (sham and 6-OHDA) were divided into three sub-groups (n = 10; vehicle (saline), Mph 3.0 mg/kg and 5.0 mg/kg). After injection, 20% of the lesioned mice died before weaning, whereas 60–80% developed hyperactivity together with dopamine (DA) depletion (Fig. 1A1–2**)**. Mice that did not meet these 2 criteria were excluded from the data analysis.

### Methylphenidate treatment for non-operant tests

**Figure 6:**
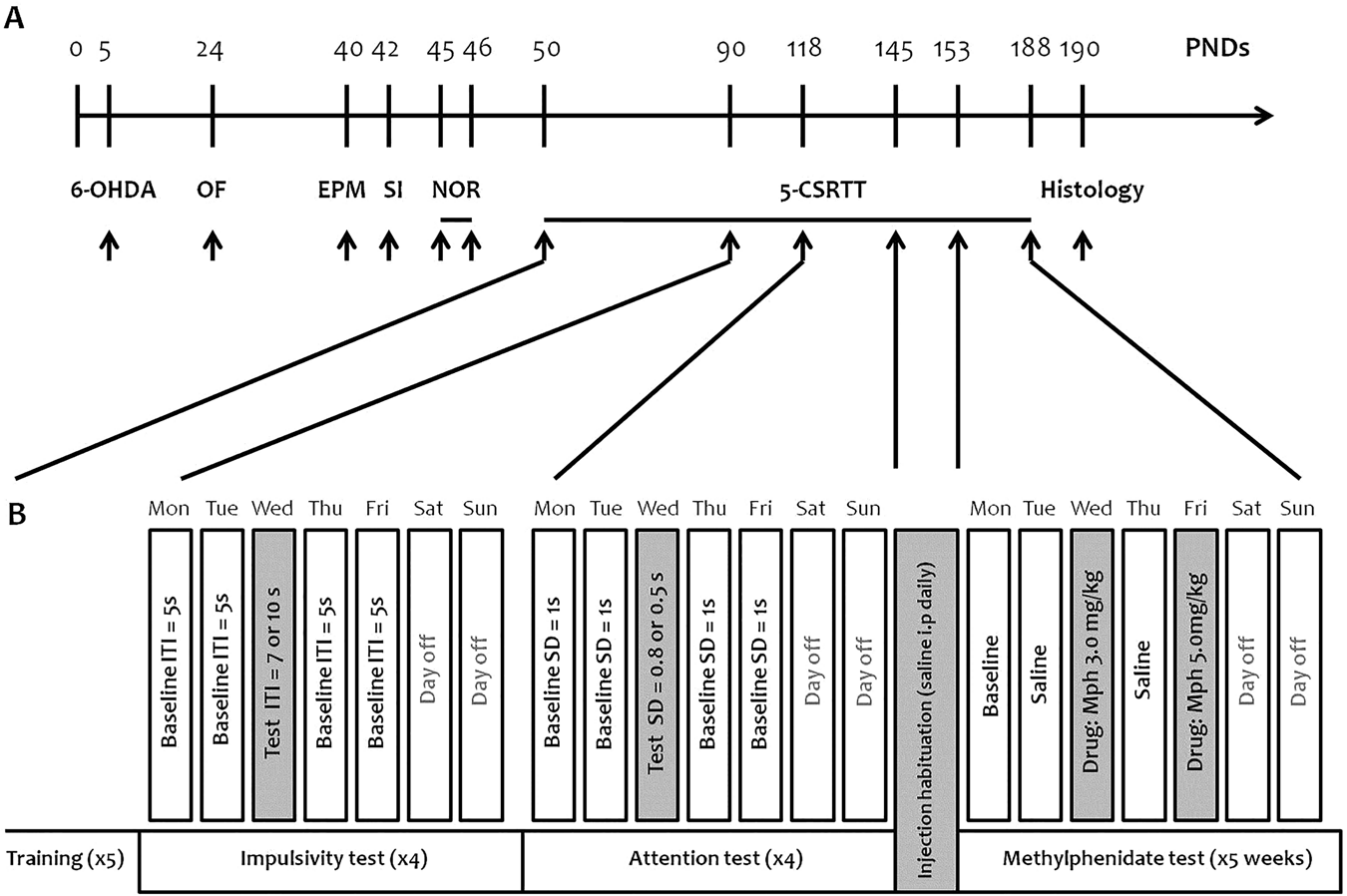
(**A**) Animal experimentation design. (**B**) Experimental design of the behavioral and pharmacological challenges in the 5-CSRTT. Upon training completion, once the animals showed a stable performance in the task, the inter-trial interval (ITI) was increased (7–10 s) and the stimulus duration (SD) was decreased (0.8–0.5 s) to challenge impulsivity and attention, respectively. After the behavioral challenge, mice were habituated to saline injections for 1 week. During the pharmacological challenge, Mph (3.0 and 5.0 mg/kg) was injected twice a week before the testing session. x4 or x5: number of weeks for training, impulsivity, attention, and methylphenidate tests.

Mph or vehicle was administered intraperitoneally (10 ml/kg in 0.9% NaCl) 45 min prior to test sessions for all the behavioral tests. The Mph doses were chosen based on previous studies^70–72^. The injection was given once before open field test, elevated plus maze test, social interaction test and then before the training day (2^nd^ day) of the novel object recognition test (Fig. 6A).

### Histology

At the end of the Mph test, in the 5-CSRTT, the saline mice of both groups (sham and 6-OHDA, n = 10 each) were used for histology. Tissue preparation, Nissl staining, Golgi Cox staining and TH Immunohistochemistry were assessed as described previously^73–75^. The slides were visualized with an Olympus microscope and images were acquired with an Olympus D72 camera.

### Behavioral tests

Spontaneous activity (open field test) was assessed in all groups at P24. From P40, behavioral and cognitive deficits were tested in all groups (see Fig. 6A): anxiety-like behavior (elevated plus maze test at P40), anti-social behavior (social interaction test at P42), short-term memory impairment (novel object recognition test at P45–46)^76–78^. For the operant test, we selected only saline mice from sham and 6-OHDA groups (n = 10, each) to evaluate attention and impulsivity (5-CSRTT training from P50)^79^. As used in various studies^80–83^, and reviewed by Robbins^84^, the percentage of correct responses, also termed response accuracy, reflects errors of commission without including errors of omission and is one of the two variables best accounting for attentional performance. The percentage of omissions (no response after stimulus presentation) is the second variable accounting for attention; it reflects detection failures. The number of premature responses is an index of impulsivity. The number of perseverative responses corresponds to another form of inhibitory deficit related to impulsive/compulsive behavior.

Upon training completion in 5-CSRTT (P90), once the animals showed a stable performance in the task, the inter-trial interval (ITI) was increased (7–10 s) and the stimulus duration (SD) was decreased (0.8–0.5 s) to challenge impulsivity and attention, respectively (Fig. 6B). Each parameter was manipulated once a week during 8 weeks: 1^st^ and 2^nd^ weeks, ITI = 7 s; 3^rd^ and 4^th^ weeks, ITI = 10 s; 5^th^ and 6^th^ weeks, SD = 0.8 s; and 7^th^ and 8^th^ weeks, SD = 0.5 s. After the behavioral challenge, mice were habituated to saline injections for 1 week. During the pharmacological challenge, Mph (3.0 and 5.0 mg/kg) were injected twice a week before the testing session for five weeks (Fig. 6B). 0.9% saline was injected i.p. on Tuesdays and Thursdays (baseline condition), while a given dose of Mph was administered 45 min before the session (3.0 mg/kg and 5.0 mg/kg on Wednesdays and Fridays, respectively). Mice were subjected to standard sessions of the 5-CSRTT with the same parameters used for the assessment of baseline responding.

### Statistical analysis

Statistical analyses were conducted using SigmaPlot 11.0 software (SigmaStat, Systat Software Inc, San Jose, CA, USA). Results were presented as mean ± standard error of the mean (SEM). The Student’s t-test was used for two-sample comparisons. For multiple sample comparisons, Two-Way ANOVA was performed, followed by Student-Newman-Keuls post-tests for behavioral analysis. Two-Way Repeated measure ANOVA followed by Holm-sidak Method post-tests was used to analyze the performance in the 5-CSRTT during baseline, ITI and SD manipulations. For the pharmacological challenges, the mean of all sessions of each 5-CSRTT parameters taken in two different sessions (vehicle and each drug dose) was used as within-subjects factor and the lesion as the between-subjects factor. A Holm-sidak Method post-tests was used to follow-up significant main effects and interactions. Principal component analysis was performed using R software (Ade4 package) based on the 20 behavioral variables that have been measured in this study^85^.

## Electronic supplementary material

supplementary data
