# Supplementary material for: Neonatal 6-OHDA lesion model in mouse induces Attention-Deficit/ Hyperactivity Disorder (ADHD)-like behaviour
Source: Sci Rep. 2018 Oct 18;8:15349. doi: 10.1038/s41598-018-33778-0 (PMC6193955; doi:10.1038/s41598-018-33778-0)
Supplement: Supplementary file 1 — supplementary data [file 41598_2018_33778_MOESM1_ESM.pdf]

Neonatal 6-OHDA lesion model in mouse induces Attention-Deficit/ Hyperactivity Disorder (ADHD)-like behaviour.

Otmane Bouchatta[1,2,3], Houria Manouze[1], Rabia Bouali-benazzouz[2,3], Nóra Kerekes[4], Saadia Ba M'Hamed[1], Pascal Fossat[2,3], Marc Landry[2,3]*, Mohamed Bennis[1]*

[1]. Laboratory of Pharmacology, Neurobiology and Behavior (URAC-37), Faculty of Sciences, Cadi Ayyad University, Marrakesh, Morocco.

[2]. Bordeaux University, Bordeaux, France.

[3]. Interdisciplinary Institute of Neuroscience, CNRS UMR 5297, Centre Paul Broca-Nouvelle Aquitaine, Bordeaux, France.

[4]. Department of Health Sciences, University West, Trollhättan, Sweden.

*. Co-senior authors.

Correspondence: Marc Landry

E-mail: marc.landry@u-bordeaux.fr

Current address:
Université de Bordeaux,
IINS, CNRS UMR 5297
Centre Broca-Nouvelle Aquitaine
146, rue Léo Saignat,
33076 Bordeaux, France

Supplemental Methods:

1. 6-OHDA neonatal lesion at P5

10-12 females were used for the reproduction. Males were housed overnight with female in proestus stage (1: 2). Each female has 8-12 pups (4-6 male). We divided the number of male pups to sham and 6-OHDA equally. 60 mice (P5) were divided on Sham and 6-OHDA groups (n=30/each group). At P5, male pups received the norepinephrine uptake blocker desipramine hydrochloride (20 mg/kg, s.c.; Sigma-Aldrich, France). After 30 min, pups were anesthetized by hypothermia (placed on ice for 1 min) and then received 25 µg of 6-OHDA hydrobromide (Sigma-Aldrich, France) dissolved in 3 µl of ascorbic acid 0.1% into one of the lateral ventricles, at 1.5 µl/min. Sham mice received vehicle. Injections were performed manually by penetrating the skull with a 30G needle (Carpule, Bayer; Osaka, Japan) coupled to a 25 µl Hamilton syringe. The site of injection was determined at 0.6 mm lateral to the medial sagittal suture, 2 mm rostral to the lambda and 1.3 mm in depth from the skull. After the injection, the pups were warmed up at 37°C on a heating pad until recovery, and then randomly returned in their cages until weaning.

2. Histology

2.1. Nissl staining

Mice were deeply anesthetized with an i.p injection of urethane 40% (1g/kg, from Sigma-Aldrich, France), and perfused transcardially with phosphate buffer (100 mM, pH 7.4) followed by ice-cold 4% paraformaldehyde (wt/vol). The brains were removed and post-fixed overnight in phosphate buffer (50 mM, pH 7.4) containing 4% paraformaldehyde. Brains (n=4) were cut in 30 µm coronal sections at the level of the anterior cingulate cortex (ACC: 1.20 mm to – 0.10 mm from bregma) with a Leica Automatic Vibratome (VT-1200S) (Leica, Solms, Germany), mounted on gelatin-coated microscope slides and left to dry. Nissl staining (0,2% cresyl violet, 0,5% acetic acid, 0,01 M sodium acetate in sterile water) was performed for 20 minutes at 37 °C. Sections were dehydrated in ethanol 70%, 95%, 100%, xylene and mounted with Eukitt (Sigma-Aldrich). Thickness of the total cortex and of its layers in sham and 6-OHDA mice was analyzed on 3 adjacent sections. To quantify the volume of each brain region, their area (6 slices per mouse) was measured

using an Olympus Plus software and images were digitized with a high-resolution Olympus D71 camera, and were analyzed using ImageJ software.

2.2. Golgi Cox staining

Procedure. Rapid-Golgi fixative solution was prepared as described by **Patro *et al.* (2009)**[1] (5 g potassium dichromate, 5 g chloral hydrate, 8 ml glutaraldehyde, 6 ml formaldehyde, 10 drops of dimethyl-sulphoxide) in a final volume made up to 100 ml with distilled water. At the end of the behavioral tests, mice were deeply anesthetized with an i.p injection of urethane 40% (1g/kg, Sigma-Aldrich, France), and perfused transcardially with phosphate buffer (100 mM, pH 7.4) followed by ice-cold 4% paraformaldehyde (wt/vol). The brains (n = 3 mice/group) were directly immersed in the Golgi fixative solution and kept in dark amber bottles, undisturbed for 2 days, followed by a second change of the fixative for next 2 days. On the 5th day, tissues were rinsed briefly (2-3 times) with 0.75% aqueous solution of silver nitrate and kept submerged in the same solution for 2 days in dark. The tissues were again rinsed with 0.75% silver nitrate solution (2-3 times) and kept in the same solution for 3 days in dark. Subsequently, tissue pieces were washed carefully in 70% alcohol to remove precipitates of silver and then the sections were cut using Leica Automatic Vibratome (VT-1200S) at 100 µm thickness. The sections were collected in 70% alcohol, dehydrated in absolute alcohol and cleared in xylene. The slides were visualized with the Olympus Plus software and images were digitized with a high-resolution Olympus D71 camera.

Quantification of Neuronal Morphology. Spine densities and dendritic length of layer II/III pyramidal neurons of the dorsal anterior cingulate cortex (ACd) were measured. The ACd was defined according to **Uylings *et al.* (2003)**[2] and the commonly used nomenclature of **Krettek *and* Price (1977)**[3]. This area corresponds to the Cg1 of **Paxinos *and* Franklin, (2001)**[4]. Neurons for analysis had to satisfy the following criteria: (i) location of soma within layer II/III and within the boundaries of the ACd, (ii) complete staining of apical and basal dendritic trees within the 150-µm section, (iii) morphology of primary apical dendrites branches. Neurons with longer primary apical dendrites possessing oblique dendrites, which typically are found deeper in layer III, were not included in this study.

Dendritic diameter and complexity. A Z-series of images from 10 neurons (each mouse) were collected every 1 µm at 60X magnification and analyzed using ImageJ software. The

diameter of the apical dendrite was measured at 10 or 100 microns from the soma. For each neuron, the numbers of thin, stubby and mushroom spines[5] were evaluated in 2 segments of 50 µm of secondary basal dendrites or primary and secondary apical dendrites.

Spine frequency. All protrusions, thin or stubby, with or without terminal bulbous expansions, were counted as spines if they were in direct continuity with the dendritic shaft. The average spine frequency [number of visible spines per µm of dendritic length] was calculated for the complete dendrite by dividing the number of spines by the dendritic length. The results were expressed as mean spine frequencies per hemisphere (HEM). No attempt was made to correct for hidden spines[6], since the use of visible spine counts for comparison between different experimental conditions had been validated previously[7]. All measurements were made by an experimenter blind to the experimental conditions of the animals.

2.3. TH immunohistochemistry

Briefly, adult mice were transcardiacally perfused with 4% paraformaldehyde. Brains (n=3) were removed, postfixed and cryoprotected. Coronal brain sections of the midbrain and striatum (30 µm) were collected using a freezing sliding microtome (Leica SM2000R, Germany), incubated with a rabbit polyclonal anti-TH antiserum (Chemicon International, CA, USA) and developed with a biotinylated anti-rabbit IgG followed by an avidin-biotin-peroxidase complex (Vector Laboratories, CA, USA). The peroxidase activity was revealed with diaminobenzidine. TH-positive neurons were counted at the level of the midbrain in at least four sections per mouse.

3. Behavioral tests

3.1. Open field (OF) test

The OF test was used to assess the general locomotor activity. It consisted of a square black box made of Plexiglas (50×50×50 cm high), equipped with the video-based Ethovision video tracking System (Noldus Information Technology, Wageningen, The Netherlands). Each animal was placed in the box for ten minutes while behavior was recorded according to published protocols[8,9]. The distance traveled in the box was

measured, as well as the velocity and the amount of time of animal movement. The box was cleaned with 70% ethanol between each test.

3.2. Elevated Plus Maze (EPM) test

The EPM measures the anxiety induced by open spaces, as well as height. The apparatus comprised two opposing open arms (50 × 5 cm) and two closed arms (50 × 5 × 15 cm), which joined at a square central area (5 × 5 cm) to form a plus sign[10]. The entire apparatus was elevated to a height of 45 cm above the floor. All mice were placed in the center of the maze and were allowed to run freely around the maze for 5 minutes with their behavior being recorded using a video tracking system made of a CCD video camera above the maze. After each test, the mice were returned to their home cage, and the plus-maze was cleaned with a water-moistened paper towel and dried after each mouse. For this test, the time spent and the number of entries into each arm were analyzed automatically using EthoVision software (Noldus Information Technology).

3.3. Novel Object Recognition (NOR) test

The NOR test assesses short term memory by testing the mouse ability to remember whether it had previously encountered an object or not[11]. It is based on the fact that a mouse will spend more time investigating and exploring an object which it has never seen (novel object), compared to an object it has encountered before (familiar object). The open field arena described above was used for the novel object recognition test. Mice were habituated to the box a day before the test. On the test day, mice were placed in the box for the acquisition period with two identical objects, and were allowed to explore and familiarize themselves with the objects for 5 minutes, and then returned to their home cages for 60 min inter-trial interval (ITI) period. Then, they were placed back in the box for the test recognition. Everything was the same as during the acquisition period, except that one of the two identical objects was replaced by a novel object. During the testing period, mice were allowed to explore both objects for 5 minutes. The time spent exploring each object was recorded with a video camera, and scored automatically using EthoVision software (Noldus Information Technology). We measured the time ratio spent on each object (familiar or novel) as: [Tr = time spent on an object/total time spent on both objects] and the discrimination ratio as: [Dr = (novel object exploration time/total

exploration time of the two objects) – (familiar object exploration time/total exploration time of two objects)].

3.4. Social Interaction (SI) test in a novel environment

The SI test is a simple test in which behaviors are video-recorded and analyzed to assess active interaction time between the tested (experimental) mouse and a novel (stimulus) mouse placed together into the test box (40 cm × 40 cm × 30 cm) and allowed to explore freely for 10 min. At the beginning of the social contact test, the experimental and stimulus mice were placed at opposite corners of the arena, facing away from each other. The two animals were differentiated by marking the backs or tails of the stimulus mice with an indelible marker. The mice in every pair were matched for age, gender and weight. Social behavior was monitored by a CCD camera, which was connected to a computer. Data acquisition and analysis were performed automatically using EthoVision software (Noldus Information Technology). The amount of contacts was measured in accordance with a previously published protocol[12]. Counts were made of the total number of escapes and approaches between the two animals during the total testing period (10 min). The dependent measure was the ratio (R) between weighted movement 'from' (used as an objective measure for the intensity of avoidance) and weighted movement 'to' (used as an objective measure for the intensity of approach). An approach was defined as the nose-point of the experimental animal in proximity relative to the body points of the stimulus animal, with the experimental animal considered to be exploring or sniffing the other animal. The total duration of contacts, the agonistic behavior delivered defined as 'dominant behavior'[13], and the first attack latency were also measured.

3.5. Five-Choice Serial Reaction Time Task (5-CSRTT) test

In the beginning, all animals had restricted access to food and their body weights were adjusted to 85% of their free-feeding weights. Animals had free access to water at all times except during training sessions.

Apparatus. Mice were trained in operant chamber (24×20×15 cm) placed inside ventilated sound-attenuating cubicles (Med Associates Inc., St. Albans, VT, USA). The chamber consisted of a curved wall containing nine round apertures equipped with infrared

detectors and bright white led (1.7 W) at the rear. Four of the nine apertures were blocked with a metal plate, thus allowing five functioning apertures equally spaced 2.5 cm apart. A magazine was located centrally in the opposite wall, equipped with an infrared detector and connected to a pellet dispenser delivering 25 mg food pellets into a food tray covered by a hinged door (López Morenas, SL, Spain). Each chamber was automatically controlled by Packwin software (Panlab S.P., Cornella, Barcelona, Spain) and data were collected via a computer.

Training procedure. Adaptation of the 5-CSRTT to the mouse was first reported by Humby et al. (1999). Subsequently, a number of reports have documented procedures for training mice in this task. Overall, the training protocols reported in these studies are similar to the original procedure[14]. The behavioral training was carried out during the light phase. The training consisted of a 20-min daily session for 5 days a week over a period of 20 weeks. All sessions in the 5-CSRTT were conducted with the house light of the apparatus extinguished[15]. Pre-training and training procedures were identical to previous studies[14,16,17,18,19,20].

Impulsivity and attention tests. Upon training completion, once the animals showed a stable performance in the task, impulsivity and attention were challenged with increasing the inter-trial interval (ITI) (7–10 s) and decreasing the stimulus duration (SD) (0.8–0.5 s), respectively (**Fig. 6B**). Each parameter was manipulated once a week during 8 weeks. After the behavioral challenge, mice were habituated to saline injections for 1 week. During the testing weeks, Mph (3.0 and 5.0 mg/kg) was injected twice a week before the session (**Fig. 6B**). Mice were then subjected to standard 5-CSRTT sessions with the same parameters used for the assessment of baseline responding.

For each session, the following variables were analyzed: the number of trials completed (number of correct responses + incorrect responses), the percentage of correct responses ([number of correct responses/number of trials completed]×100), the percentage of omissions ([omissions/trials started that were not terminated by a premature response]×100), the percentage of premature responses ([premature responses/trials started]×100), the percentage of perseverative responses ([perseverative responses/correct responses]×100). As reviewed by **Robbins (2002)**[16], the percentage of correct responses, also termed response accuracy in other studies, reflects errors of

commission without including errors of omission and is one of the two variables best accounting for attentional performance. The percentage of omissions (no response after stimulus presentation) is the second variable accounting for attention; it reflects detection failures. The percentage of premature responses is an index of impulsivity. The percentage of perseverative responses corresponds to another form of inhibitory deficit related to compulsive behavior.

4. Principal Component Analysis (PCA)

Briefly, the contribution of each variable to the variance in the first and second components of the PCA was calculated. In a second step, the location of each animal group was graphically represented on the plane defined by the first and second components of the PCA. The separation between pairs of groups was evaluated by calculating the inertia, which was defined as the ratio of the between-group variance to the global variance. The statistical significance of inertia for group separation was estimated using a Monte Carlo permutation test (1000 runs). In each run, the simulated inertia was calculated. The distribution of simulated inertia values was then compared to the real inertia. A p-value was then calculated as the ratio of the number of simulations in which the simulated inertia was larger than the real inertia to the total number of runs and fixed to $p<0.01$.

Supplemental References:

1. Patro, N., Shrivastava, M., Tripathi, S., Patro, I.K. S100β upregulation: a possible mechanism of deltamethrin toxicity and motor coordination deficits. *Neurotoxicol. Teratol*. 31, 169-176; 10.1016/j.ntt.2008.12.001 (2009).
2. Uylings, H.B., Groenewegen, H.J., Kolb, B. Do rats have a prefrontal cortex? *Behav. Brain. Res.* 146(1), 3-17 (2003).
3. Krettek, J.E., Price, J.L. Projections from the amygdaloid complex to the cerebral cortex and thalamus in the rat and cat. *J. Comp. Neurol.* 172(4), 687-722 ; 10.1002/cne.901720408 (1977).
4. Paxinos, G., Franklin, K.B.J. The Mouse Brain in Stereotaxic Coordinates, 2nd edition. Academic Press, San Diego, California, USA, pp. 216 (2001).
5. Harris, K.M., Jensen, F.E., Tsao, B. Three-dimensional structure of dendritic spines and synapses in rat hippocampus (CA1) at postnatal day 15 and adult ages: implications for the maturation of synaptic physiology and long-term potentiation. *J. Neurosci.* 12(7), 2685-2705 (1992).
6. Feldman, M.L., Peters, A. A technique for estimating total spine numbers on Golgi-impregnated dendrites. *J. Comp. Neurol.* 188(4), 527-542; 10.1002/cne.901880403 (1979).
7. Horner, C.H., Arbuthnott, E. Methods of estimation of spine density--are spines evenly distributed throughout the dendritic field? *J. Anat.* 177, 179-84 (1991).
8. Pietropaolo, S. Mood and Anxiety-related Phenotypes in Mice: Characterization Using Behavioral Tests-Edited by TD Gould. *Genes. Brain. Behav.* 9(5), 544-544 (2010).
9. Hart, P.C., Bergner, C.L., Smolinsky, A.N., Dufour, B.D., Egan, R.J., LaPorte, J.L., Kalueff, A.V. Experimental models of anxiety for drug discovery and brain research. *Methods. Mol. Biol.* 602, 299-321; 10.1007/978-1-60761-058-8_18 (2010).
10. Handley, S.L., McBlane, J.W. 5-HT drugs in animal models of anxiety. *Psychopharmacology*. 112(1), 13-20 (1993).
11. Ennaceur, A., Delacour, J. A new one-trial test for neurobiological studies of memory in rats. 1: Behavioral data. *Behav. Brain. Res.* 31(1), 47-59 (1988).
12. Lapiz-Bluhm, M.D.S., Bondi, C.O., Doyen, J., Rodriguez, G.A., Bédard-Arana, T., Morilak, D.A. Behavioural assays to model cognitive and affective dimensions of

depression and anxiety in rats. *J. Neuroendocrinol.* 20, 1115-1137; 10.1111/j.1365-2826.2008.01772.x (2008).

13. Clipperton-Allen, A.E., Almey, A., Melichercik, A., Allen, C.P., Choleris, E. Effects of an estrogen receptor alpha agonist on agonistic behaviour in intact and gonadectomized male and female mice. *Psychoneuroendocrinology*. 36, 981-995; 10.1016/j.psyneuen.2010.12.010 (2011).
14. Humby, T., Laird, F.M., Davies, W., Wilkinson, L.S. Visuospatial attentional functioning in mice: interactions between cholinergic manipulations and genotype. *Eur. J. Neurosci.* 11, 2813-2823 (1999).
15. Humby, T., Wilkinson, L., Dawson, G. Assaying aspects of attention and impulse control in mice using the 5-choice serial reaction time task. *Curr. Protoc. Neurosci.* 8-5; 10.1002/0471142301 (2005).
16. Robbins, T. The 5-choice serial reaction time task: behavioural pharmacology and functional neurochemistry. *Psychopharmacology*. 163, 362-380; 10.1007/s00213-002-1154-7 (2002).
17. Oliver, Y.P., Ripley, T.L., Stephens, D.N. Ethanol effects on impulsivity in two mouse strains: similarities to diazepam and ketamine. *Psychopharmacology*. 204, 679-692; 10.1007/s00213-009-1500-0 (2009).
18. Moreno, M., Cardona, D., Gómez, M.J., Sánchez-Santed, F., Tobeña, A., Fernández-Teruel, A., Campa, L., Suñol, C., Escarabajal, M.D., Torres, C., Flores, P. Impulsivity characterization in the Roman highand low-avoidance rat strains: behavioral and neurochemical differences. *Neuropsychopharmacology*. 35, 1198–208; 10.1038/npp.2009.224 (2010).
19. Harati, H., Majchrzak, M., Cosquer, B., Galani, R., Kelche, C., Cassel, J.C., Barbelivien, A. Attention and memory in aged rats: impact of lifelong environmental enrichment. *Neurobiol. Aging*. 32, 718-736; 10.1016/j.neurobiolaging.2009.03.012 (2011).
20. Reverte, I., Peris-Sampedro, F., Basaure, P., Campa, L., Suñol, C., Moreno, M., Domingo, J.L., Colomina, M.T. Attentional performance, impulsivity, and related neurotransmitter systems in apoE2, apoE3, and apoE4 female transgenic mice. *Psychopharmacology*. 233, 295-308; 10.1007/s00213-015-4113-9 (2016).

Supplementary figure legends

Figure S1: Spontaneous locomotor activity at P40. **A.** Distance traveled during 10 min. **B.** Mobility mean time. **C.** Animal velocity. All data shown are means ± SEM, n= 10 mice per group, *$p<0.05$ and **$p<0.01$ *vs* sham.

Figure S2: Baseline performance of sham and 6-OHDA mice in the 5-CSRTT (SD = 1 s) for the last four successive days (X axis) of training. **A.** Percentage of accuracy. **B.** Percentage of omissions. **C.** Percentage of premature responding. **D.** Percentage of perseverative responses. Sham mice were more accurate than 6-OHDA mice, and made omission errors at lower rate. By contrast, 6-OHDA mice were less accurate than sham mice, made more omission errors, premature and perseverative responses. Our findings suggest that attentiveness may be impaired and impulsivity increases in 6-OHDA mice. All data shown are means ± SEM. from 10 mice per group, ***: $p<0.001$.

Figure S3: The envelopes of selected groups from the same PCA are presented separately in order to facilitate comparisons. **A**. Control, Sham and 6-OHDA groups. **A1.** Control and sham groups are statistically equivalent. **A2.** 6-OHDA group is significantly different from sham group. **B.** Methylphenidate (Mph) treatment on 6-OHDA mice. **B1.** Mph shifted groups toward left along the x-axis, showing up a partial recovery from ADHD symptoms. **B2.** Mph group (5 mg) is significantly different from 6-OHDA group. **C.** Mph treatment on sham group. **C1.** Groups are shifted groups towards left on x-axis and down on y-axis, revealing that Mph worsen symptoms of ADHD in control groups. **C2.** Groups that receive Mph 5mg are statistically different from control groups.

Table S1. Strength of each variable analyzed in the principal component analysis regarding the 2 components.

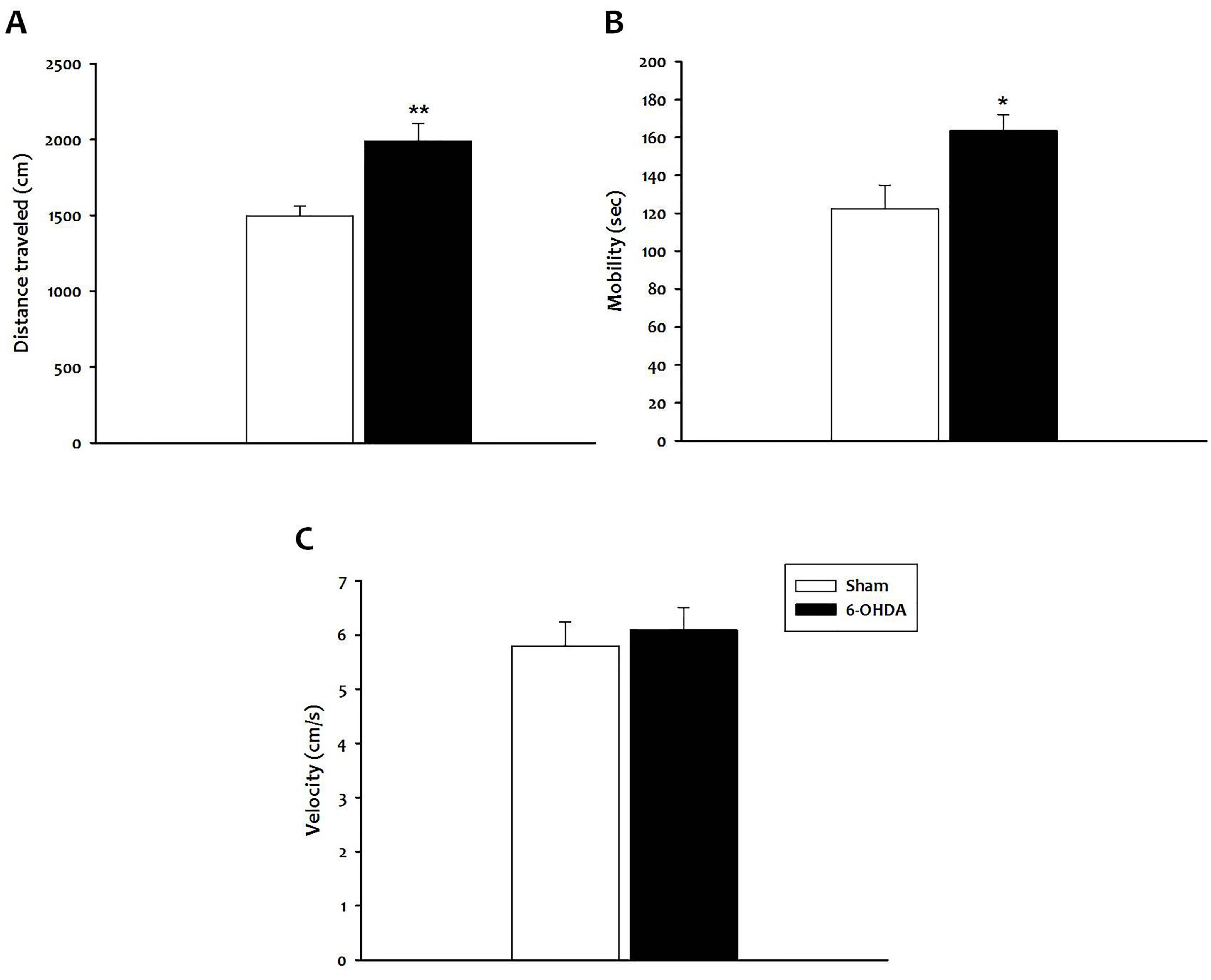

A
Distance traveled (cm)
2500
2000
1500
1000
500
0
**
B
Mobility (sec)
200
180
160
140
120
100
80
60
40
20
0
*
C
Velocity (cm/s)
7
6
5
4
3
2
1
0
Sham
6-OHDA

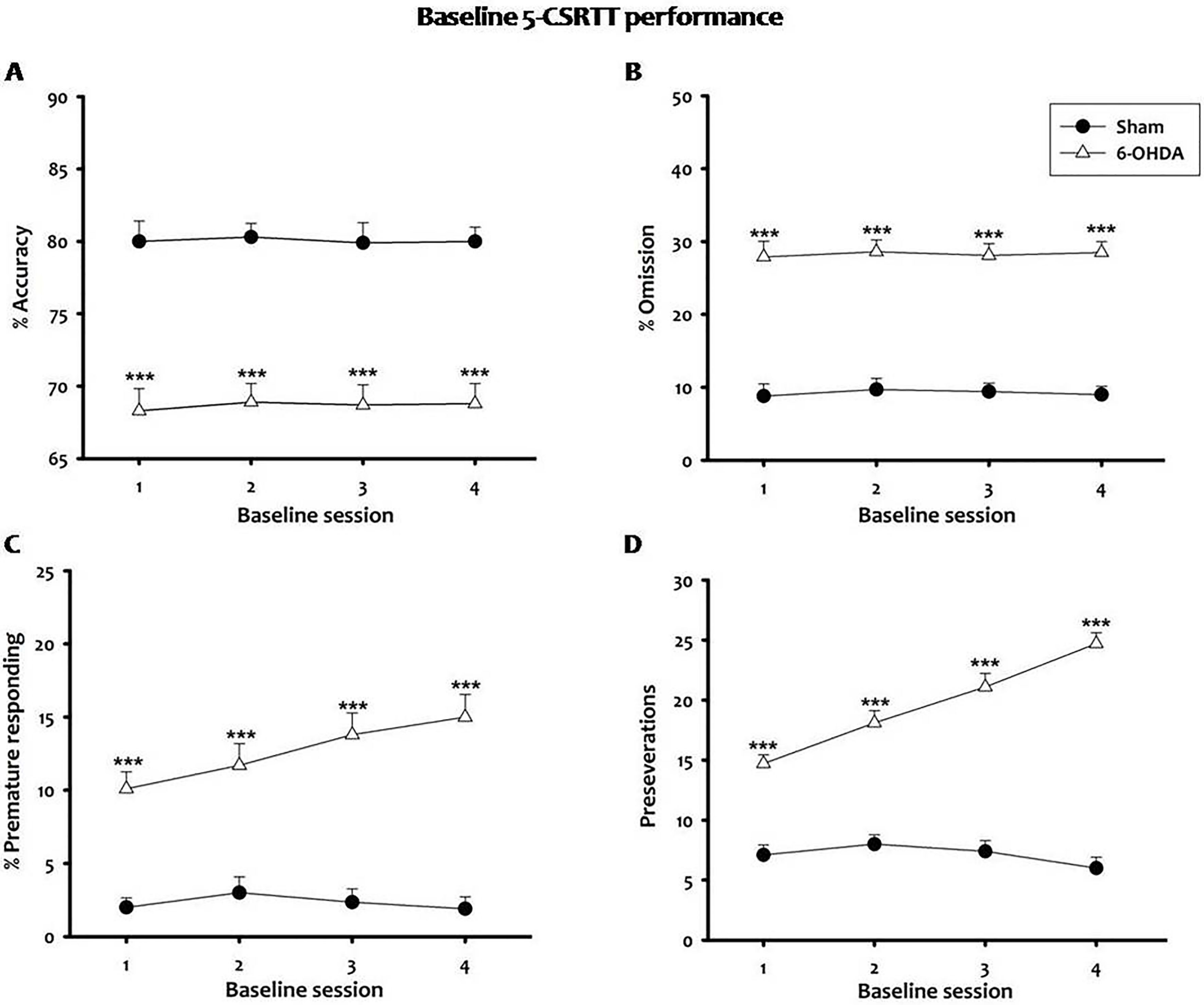

Baseline 5-CSRTT performance
A
% Accuracy
90
85
80
75
70
65
***
1
2
3
4
Baseline session
B
% Omission
50
40
30
20
10
0
Sham
6-OHDA
C
% Premature responding
25
20
15
10
5
D
Preseverations

Figure S3

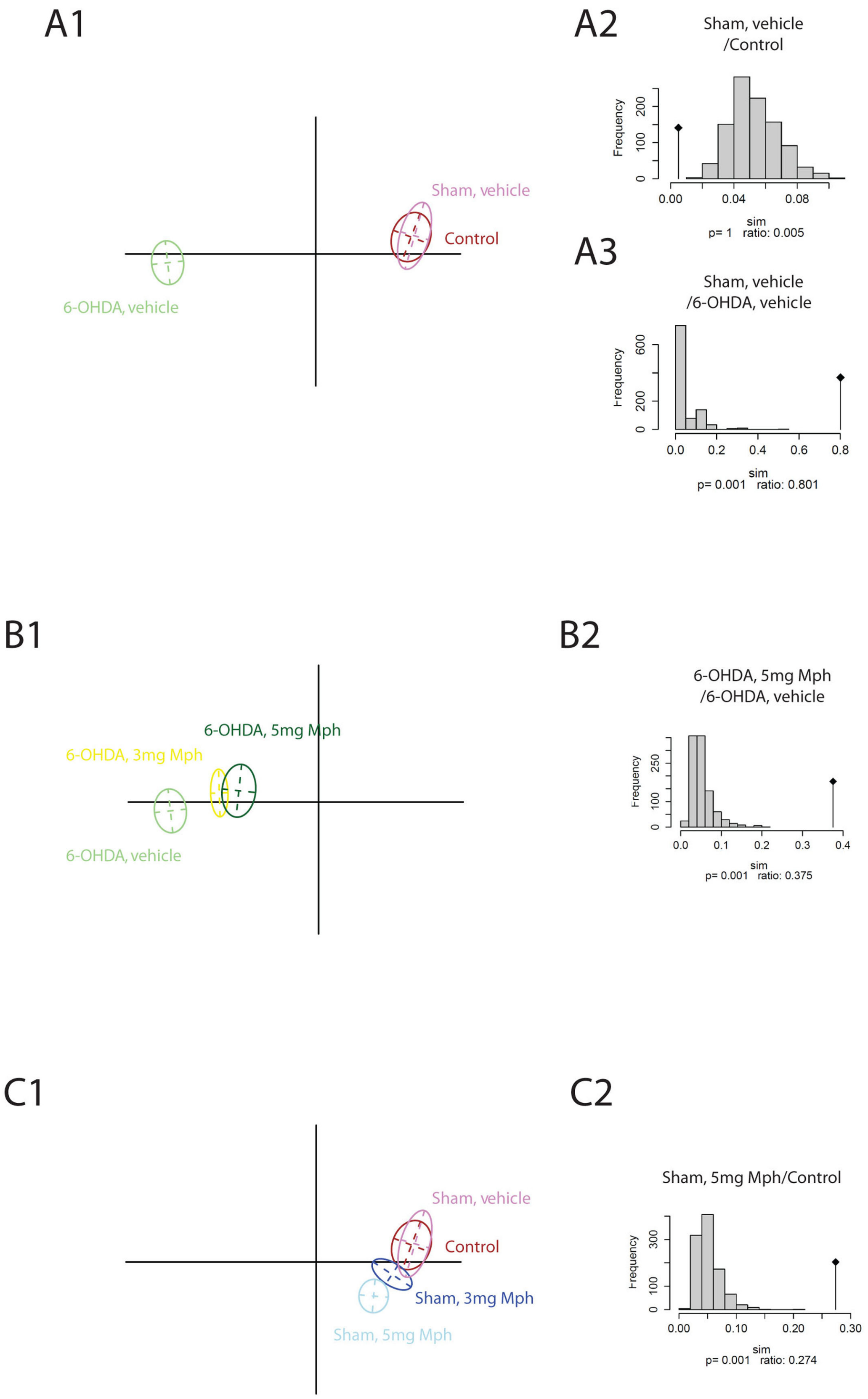
A1
Sham, vehicle
Control
6-OHDA, vehicle
A2
Sham, vehicle
/Control
Frequency
0
100
200
0.00
0.04
0.08
sim
p= 1 ratio: 0.005
A3
Sham, vehicle
/6-OHDA, vehicle
Frequency
0
200
600
0.0
0.2
0.4
0.6
0.8
sim
p= 0.001 ratio: 0.801
B1
6-OHDA, 5mg Mph
6-OHDA, 3mg Mph
6-OHDA, vehicle
B2
6-OHDA, 5mg Mph
/6-OHDA, vehicle
Frequency
0
100
250
0.0
0.1
0.2
0.3
0.4
sim
p= 0.001 ratio: 0.375
C1
Sham, vehicle
Control
Sham, 3mg Mph
Sham, 5mg Mph
C2
Sham, 5mg Mph/Control
Frequency
0
100
300
0.00
0.10
0.20
0.30
sim
p= 0.001 ratio: 0.274

Tabls S1

	Variables	Comp1	Comp2
	Distance	**644**	0
Hyper-	Mobility	374	**1296**
activity	Speed	**574**	188
	Open duration	331	**1148**
	Open number	323	**1227**
Anxiety	Open latency	**517**	**1533**
	Close duration	299	**789**
	Close number	54	432
	Total duration of contact	**647**	1
	Mean duration of contact	**632**	7
Aggression	First attack latency	**668**	76
	Weighted to	**654**	10
	Agonistic behavior	**663**	3
	Acquisition trial	**542**	120
Memory	Test trial	456	**2049**
	Discrimination ratio	470	329
Attention	Accuracy	423	203
	Omission	**671**	151
Impulsivity	Performance responding	**609**	1
	Perserveration	448	439
